# Intrathecal Th17- and B cell-associated cytokine and chemokine responses in relation to clinical outcome in Lyme neuroborreliosis: a large retrospective study

**DOI:** 10.1186/s12974-017-0789-6

**Published:** 2017-02-01

**Authors:** Paula Gyllemark, Pia Forsberg, Jan Ernerudh, Anna J. Henningsson

**Affiliations:** 1 Department of Infectious Diseases, Region Jönköping County, SE-551 85 Jönköping, Sweden; 2 Department of Clinical and Experimental Medicine and Department of Infectious Diseases, Linköping University, Linköping, Sweden; 3 Department of Clinical and Experimental Medicine and Department of Clinical Immunology and Transfusion Medicine, Linköping University, Linköping, Sweden; 4 Clinical Microbiology, Division of Medical Services, Jönköping, Region Jönköping County Sweden

**Keywords:** Lyme neuroborreliosis, Cerebrospinal fluid, Cytokines, Chemokines, APRIL, BAFF, CCL20, CXCL 1, CXCL13, IL-17A

## Abstract

**Background:**

B cell immunity, including the chemokine CXCL13, has an established role in Lyme neuroborreliosis, and also, T helper (Th) 17 immunity, including IL-17A, has recently been implicated.

**Methods:**

We analysed a set of cytokines and chemokines associated with B cell and Th17 immunity in cerebrospinal fluid and serum from clinically well-characterized patients with definite Lyme neuroborreliosis (group 1, *n* = 49), defined by both cerebrospinal fluid pleocytosis and *Borrelia*-specific antibodies in cerebrospinal fluid and from two groups with possible Lyme neuroborreliosis, showing either pleocytosis (group 2, *n* = 14) or *Borrelia*-specific antibodies in cerebrospinal fluid (group 3, *n* = 14). A non-Lyme neuroborreliosis reference group consisted of 88 patients lacking pleocytosis and *Borrelia*-specific antibodies in serum and cerebrospinal fluid.

**Results:**

Cerebrospinal fluid levels of B cell-associated markers (CXCL13, APRIL and BAFF) were significantly elevated in groups 1, 2 and 3 compared with the reference group, except for BAFF, which was not elevated in group 3. Regarding Th17-associated markers (IL-17A, CXCL1 and CCL20), CCL20 in cerebrospinal fluid was significantly elevated in groups 1, 2 and 3 compared with the reference group, while IL-17A and CXCL1 were elevated in group 1. Patients with time of recovery <3 months had lower cerebrospinal fluid levels of IL-17A, APRIL and BAFF compared to patients with recovery >3 months.

**Conclusions:**

By using a set of markers in addition to CXCL13 and IL-17A, we confirm that B cell- and Th17-associated immune responses are involved in Lyme neuroborreliosis pathogenesis with different patterns in subgroups. Furthermore, IL-17A, APRIL and BAFF may be associated with time to recovery after treatment.

## Background

Lyme neuroborreliosis (LNB) is the dominating disseminated form of Lyme borreliosis in Sweden [1] as well as in Europe [2]. The pathogenesis of LNB involves a complex immune response with an initial innate response elicited by *Borrelia burgdorferi* (*B.b.*) interacting with recognition receptors such as Toll-like receptor 2, subsequently resulting in activation and recruitment of B and T cells to the central nervous system (CNS). The chemokine C-X-C motif ligand (CXCL)13 is a key molecule in B cell recruitment to the CNS [3], and several studies have shown high concentrations of CXCL13 in the cerebrospinal fluid (CSF) in both children and adults with LNB [4–6]. CXCL13 is postulated to be a diagnostic marker in acute LNB since it may be elevated in CSF before intrathecally produced *B.b.*-specific antibodies can be detected; however, different cutoff levels have been discussed, e.g. 142 and 250 pg/mL, respectively [4–8]. The cytokines a proliferation-inducing ligand (APRIL) and B cell activating factor (BAFF) are important in B cell development and survival [9], and raised CSF levels have been detected in other neuroinflammatory conditions [10, 11]. Although increased BAFF levels in CSF have been reported in LNB [12], the relative contribution of B cell-associated factors such as CXCL13, APRIL and BAFF in LNB inflammation and clinical outcome is mainly unknown.

Recent studies have indicated involvement of T helper (Th)17 cells in the intrathecal immune response in patients with LNB [13–15]. IL-17A, a cytokine produced by Th17 cells, is a potent activator of neutrophils in defeating extracellular microbes, but its wider role in the pathogenesis and clinical outcome of LNB is unclear [13, 16, 17]. CXCL1 (previously known as growth regulated oncogene-α, GRO-α), a neutrophil recruiting chemokine, and CCL20 (macrophage inflammatory protein-3α, MIP-3α) a Th17 recruiting chemokine, are both induced by Th17 cells [17]. While elevated IL-17A levels in CSF have been reported in LNB [13–15], information on its potential association with clinical outcome is still lacking, and it is not known if chemokines downstream of Th17 are increased in LNB.

A basic understanding of the molecules involved in the pathogenesis is a prerequisite for the identification of prognostic biomarkers and in the long run for finding potential therapeutic targets. The aims of this study were to evaluate the putative involvement of Th17- and B cell-associated immune response and to assess associations with disease course in LNB by analysing IL-17A and its downstream chemokines CCL20 and CXCL1, as well as B cell-associated factors APRIL, BAFF and CXCL13.

## Methods

### Patients

We included retrospectively 165 patients in Jönköping County, Sweden, who had been investigated by lumbar puncture (LP) and blood sampling during 2007–2009 to verify or exclude suspected LNB. Medical records were scrutinized, and the patients were divided into four groups based on the CSF findings (see Tables 1, 2 and 3 for demographic and clinical characteristics) and in accordance with the European Federation of Neurological Societies (EFNS) guidelines [18]. Patients in group 1 (definite LNB, *n* = 49) had both CSF pleocytosis and *Borrelia*-specific antibodies in CSF. Group 2 (possible LNB pleocytosis, *n* = 14) had symptoms strongly suggestive of LNB, short duration of symptoms and CSF pleocytosis but not (yet) *Borrelia*-specific antibodies in CSF. Group 3 (possible LNB Ab^+^, *n* = 14) had *Borrelia*-specific antibodies in CSF, but no pleocytosis and symptoms were less suggestive of LNB. As a non-LNB reference group, we selected 88 gender- and age-matched patients from the same cohort investigated for suspected LNB 2007–2009, in whom LNB was excluded based on no *Borrelia*-specific serum or CSF antibodies, no CSF pleocytosis and normal CSF-albumin. The reference group consisted of patients where the LP was part of a neurological investigation and in whom no neurological diagnosis was verified (*n* = 56) or they later received other neurological diagnoses such as Bell’s palsy (*n* = 18) or Alzheimer’s disease, Parkinson’s disease and stroke (*n* = 14).

**Table 1:** Characteristics of the four study groups

	Group 1 Definite LNB *n* = 49	Group 2 Possible LNB pleocytosis *n* = 14	Group 3 Possible LNB Ab^+^ *n* = 14	Group 4 Non-LNB patients *n* = 88	
*Borrelia*-specific AI or *Borrelia*-specific antibodies in CSF	+	–	+		–
CSF pleocytosis	+	+	–		–
*Borrelia*-specific IgG/IgM antibodies detected in serum, *n* (%)	43 (88)	11 (79)	8 (57)		0 (0)
CSF-albumin/S-albumin median (range)	16 (3.4–69)***	5.8 (2.7–45)*	5.7 (2.4–17)		4.0 (1.5–9.5)
IgG-index median (range)	0.7 (0.0–2.6)***	0.6 (0.4–0.8)***	0.5 (0.4–0.6)		0.5 (0.0–0.6)
Men *n* (%)	29 (59)	9 (64)	8 (57)		39 (44)
Women *n* (%)	20 (41)	5 (36)	6 (43)		49 (56)
Median age years (range)	32 (4–72)	8.5 (3–39)**	62 (32–82)*		23 (1–83)
Median duration symptoms before LP weeks (range)	2.0 (0.1–104)	0.5 (0.1–3.0)*	2.0 (0.1–156)		4.0 (0.1–520)
Head/neck pain *n* (%)	32 (65)	6 (43)	8 (57)		27 (31)
Cranial nerve palsy *n* (%)	22 (45)	9 (64)	0 (0)		20 (23)
Radiculitis *n* (%)	20 (41)	2 (14)	1 (7)		0 (0)
Patients with duration of symptoms after treatment under 3 months *n* (%)	40 (87)^a^	8 (57)	6 (67)^b^		–

*Pleocytosis*: >5 mononuclear cells/mL CSF

*LNB* Lyme neuroborreliosis, *n* number of patients, *Ab* ^*+*^ antibody, *AI* antibody index, *CSF* cerebrospinal fluid, *S* serum, *LP* lumbar puncture

*Significant difference compared to the non-LNB group (group 4). *p < 0.01, **p < 0.001, ***p < 0.0001

^a^Group 1: information was lacking in 3 patients, thus total *n* = 46

^b^Group 3: five patients did not receive any treatment, thus total *n* = 9

**Table 2:** Characteristics of clinical parameters in group 2 (possible LNB pleocytosis)

Pat. Nr.	Symptoms before LP	Head/neck	Fatigue	Fever	Vertigo	Radiculitis	Cranial nerve	Other symptoms
	Weeks	Pain					Palsy	
1	0.7						x	
2	0.3						x	
3	1.6	x					x	
4	0.4	x	x					
5	1.0					x		
6	0.6						x	
7	0.4						x	
8	0.1	x	x				x	
9	0.4						x	
10	0.3		x				x	
11	3.0	x	x					
12	0.7	x	x		x			
13	1.0	x	x	x		x		
14	0.3			x			x	

*LNB* Lyme neuroborreliosis, *Pat. Nr.* patient number, *LP* lumbar puncture

**Table 3:** Characteristics of clinical parameters in group 3 (possible LNB Ab+)

Pat. Nr.	Symptoms before LP	Head/neck	Fatigue	Fever	Vertigo	Radiculitis	Cranial nerve	Other symptoms
	Weeks	Pain					Palsy	
1	72	x	x					
2	3	x				x		
3	156							Unilateral vision loss
4	2	x			x			
5	104							Dysarthria, dysphagia
6	52							Dysarthria, memory loss
7	7	x						
8	1	x			x			
9	2	x	x					
10	0.7							Unilateral vision loss
11	0.4	x			x			Dysarthria, vision loss
12	0.1				x			Decreased consciousness
13	2	x	x					
14	1		x					Concentration difficulties

*LNB* Lyme neuroborreliosis, *Pat. Nr.* patient number, *LP* lumbar puncture

### Serum and CSF

Serum and CSF samples were drawn prior to antibiotic treatment and stored at −20 °C.

All tests were performed at the clinical laboratory of microbiology in Jönköping. *Borrelia*-specific antibodies in serum and CSF were analysed using Lyme *Borreliosis* ELISA kit 2nd generation (Dako Cytomation, A/S, Glostrup, Denmark) between 2007 and 2008. Intrathecal antibody index (AI) was calculated using total IgG as a reference molecule [19] according to the formula: ((*Borrelia*-specific IgG in CSF (OD)/*Borrelia*-specific IgG in serum (OD))/((total IgG in CSF (mg/L)/total IgG in serum (g/L)) [20]. A *Borreli*a-specific AI >2 was indicative of intrathecal anti-*Borrelia* antibody production. From 2009, the laboratory used the IDEIA (Lyme Neuroborreliosis kit, (Dako Cytomation)). Both antibody assays use purified, native *B. afzelii* strain DK1 flagellum as test antigen, and results were interpreted according to the manufacturer’s instructions.

### Cytokine and chemokine analyses

APRIL, BAFF and CXCL13 were analysed by ELISA (Invitrogen Immunoassay Kit, KHC3051, Life Technologies, USA, and Quantikine, DBLYSOB and DCX130, (R&D) Systems, Inc., USA, respectively). IL-17A, CXCL1 and CCL20 were analysed by Luminex multiple bead technology (Milliplex Human Cytokine/Chemokine Kit, Millipore Corporation, Germany). All analyses were conducted according to the manufacturers’ instructions. The lowest detection limits were as follows: APRIL: 0.02 pg/mL, BAFF: 0.05 pg/mL in serum, 0.04 pg/mL in CSF; CXCL13: 0.04 pg/mL in serum, 0.03 pg/mL in CSF; IL-17A: 0.38 pg/mL in serum, 0.06 pg/mL in CSF; CXCL1: 3.2 pg/mL in serum, 0.14 pg/mL in CSF; CCL20: 0.29 pg/mL in serum, 0.84 pg/mL in CSF. Values under the detection limit were given half the value of the lowest point of the standard curve.

### Data handling and statistical analyses

For statistical analyses, SPSS version 20 was used. Inter-group comparisons were performed by using the non-parametrical Kruskal-Wallis test and when *p* < 0.05 followed by Mann-Whitney *U* test as a post hoc test. For children (age < 15 years), a covariance analysis has been performed with pleocytosis as a covariate. Data are given as medians and interquartile (i.q. range). For categorical variables, the chi-square test was used. Correlations were determined by Spearman’s rank order correlation. *p* values below 0.05 were considered significant.

## Results

### General description of the patients

Table 1 presents the characteristics of the different study groups. There were significant differences in age with, older individuals in group 3 compared to group 2 which consisted mainly of children. Median duration of symptoms before LP was similar in groups 1 and 3 but several days shorter in group 2. A majority of patients in groups 1 and 2 had symptom duration after treatment below 3 months. Tables 2 and 3 present symptoms and symptom duration before LP for patients in groups 2 and 3, respectively. All patients in group 2 had symptoms highly suggestive of LNB such as head or neck pain (or both), radiculitis or cranial nerve palsy. In group 3, no patients had cranial nerve palsy and only one had radiculitis and six patients had symptoms not typical for LNB, such as vision loss and dysarthria, and their duration of symptoms before LP ranged from less than a week to several years.

### B cell-associated cytokines and chemokines

**Fig. 1:**
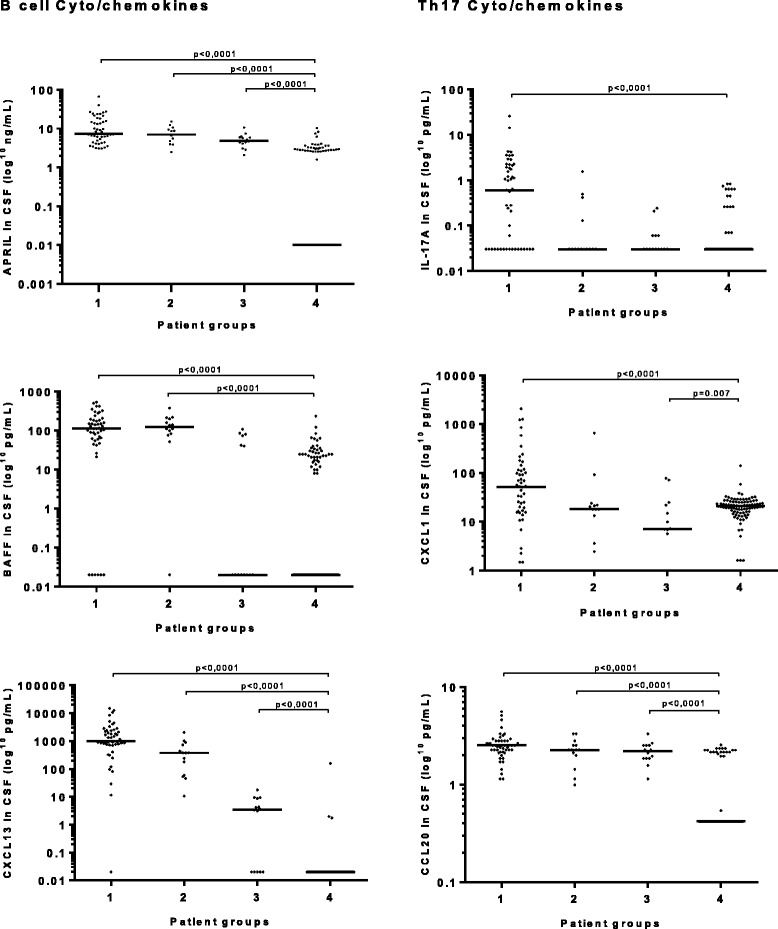
B cell- and Th17-related cytokine/chemokine levels in cerebrospinal fluid (CSF).Group 1: definite Lyme neuroborreliosis (LNB), patients with *Borrelia*-specific antibodies in CSF and pleocytosis. Group 2: possible LNB pleocytosis, patients with CSF pleocytosis but no detectable *Borrelia*-specific antibodies in CSF. Group 3: possible LNB Ab^+^, patients with *Borrelia*-specific antibodies in CSF but no pleocytosis. Group 4: non-LNB, patients without CSF pleocytosis and no detectable *Borrelia*-specific antibodies in serum or CSF. *Bars* represent the median cytokine/chemokine level in each group

CSF levels of APRIL and CXCL13 (Table 4 and Fig. 1) were significantly elevated in all LNB groups compared to the non-LNB group (group 4) while there were no differences in serum. A majority of patients in groups 1 and 2, but not in group 3, had CSF levels of CXCL13 over the previously suggested cutoff levels of 142 and 250 pg/mL (Table 5). No correlations were seen between cytokine and chemokine levels in serum and CSF.

**Table 4:** Cytokines/chemokines in serum and CSF in the four study groups

	Group 1 Definite LNB *n* = 49 Median (interquartile)	Group 2 Possible LNB pleocytosis *n* = 14 Median (interquartile)	Group 3 Possible LNB Ab^+^ n = 14 Median (interquartile)	Group 4 Non-LNB patients *n* = 88 Median (interquartile)
S-APRIL (ng/mL)	3.1 (0.8–5.7)	1.4 (0.3–1.8)	2.0 (0.5–4.4)	3.9 (0.0–5.8)
S-BAFF (pg/mL)	705 (471–857)	586 (531–873)	597 (496–763)	730 (581–935)
S-CXCL13 (pg/mL)	67 (38–99)	56 (39–84)	41 (30–58)	54 (34–81)
S-IL-17A (pg/mL)	4.0 (2.6–10)	4.5 (2.9–9.2)	5.6 (2.9–7.1)	9.8 (2.6–24)
S-CXCL1 (pg/mL)	741 (549–861)***	695 (598–1055)	599 (439–750)***	1113 (830–2425)
S-CCL20 (pg(mL)	6.1 (3.7–9.3)***	6.8 (5.3–13)***	5.2 (3.1–9.8)	0.2 (0.2–8.2)
CSF-APRIL (ng/mL)	7.4 (4.7–17)***	7.0 (4.6–9.7)***	4.9 (3.9–6.1)***	0.0 (0.0–2.9)
CSF-BAFF (pg/mL)	113 (58–191)***	125 (81–203)***	0.0 (0.0–76)	0.0 (0.0–25)
CSF-CXCL13 (pg/mL)	974 (738–2394)***	379 (57–770)***	3.4 (0.0–8.8)***	0.0 (0.0–0.0)
CSF-IL-17A (pg/mL)	0.6 (0.0–0.6)***	0.0 (0.0–0.2)	0.0 (0.0–0.1)	0.0 (0.0–0.0)
CSF-CXCL1 (pg/mL)	52 (16–117)***	16 (2.1–37)	12 (4.4–21)*	21 (16–26)
CSF-CCL20 (pg/mL)	2.5 (2.2–2.8)***	2.3 (1.9–2.6)***	2.2 (1.9–2.5)***	0.4 (0.4–0.4)

*Pleocytosis*: >5 mononuclear cells/mL CSF

*CSF* cerebrospinal fluid, *LNB* Lyme neuroborreliosis, *n* number of patients, *Ab* ^*+*^ antibody, *S* serum

*Significant difference compared to the non-LNB group (group 4). *p < 0.01, ***p < 0.0001

**Table 5:** Number of patients with CXCL13 levels in CSF over 142 and 250 pg/mL, respectively, in the four study groups

	Group 1 Definite LNB *n* = 49	Group 2 Possible LNB pleocytosis *n* = 14	Group 3 Possible LNB Ab^+^ *n* = 14	Group 4 Non-LNB patients *n* = 88
>142 pg/mL *n* (%)	43 (88)	10 (71)	0 (0)	1 (1)
>250 pg/mL *n* (%)	42 (86)	9 (64)	0 (0)	0 (0)

*Pleocytosis*: >5 mononuclear cells/mL CSF

*CSF* cerebrospinal fluid, *LNB* Lyme neuroborreliosis, *n* number of patients, *Ab* ^*+*^ antibody

### Th17-associated cytokines and chemokines

CSF levels of IL-17A, CXCL1 and CCL20 (Table 4 and Fig. 1), were all significantly elevated in group 1 compared to group 4. CCL20 was also significantly higher in groups 2 and 3 compared to group 4. In Table 4, serum levels of CXCL1 were significantly lower in groups 1 and 3 compared to group 4 whereas levels of CCL20 were significantly higher in groups 1 and 2 compared to group 4. CSF levels of IL-17A correlated with CXCL1 (rho = 0.72, *p* < 0.0001) in groups 1, 2 and 3. No correlations were seen between cytokine and chemokine levels in serum and CSF.

### Associations with demographic and clinical parameters

There were no significant differences in cytokine/chemokine levels in serum or in CSF between men and women. Regarding differences in relation to age (data not shown), we found that children <15 years of age in groups 1, 2 and 3 (*n* = 34) had significantly higher levels of BAFF (median 108 pg/mL, i.q. range 60–165, *p* < 0.001) in serum and CXCL13 in serum and CSF (77 pg/mL, 47–109, *p* = 0.001 and 920 pg/mL, 398–1706, *p* = 0.03, respectively) compared to adults. BAFF in serum also showed a strong negative correlation with age in groups 1 and 2 (rho = −0.57, *p* < 0.01). APRIL, BAFF and CXCL13 in CSF were all positively correlated with pleocytosis *(*rho = 0.51, rho = 0.51 and rho = 0.55, respectively, all *p* < 0.001). When a covariate analysis was performed with pleocytosis as a covariate, BAFF in serum was still significantly higher in children (*p* < 0.0001), but CXCL13 in serum and CSF was not. Children in groups 1, 2 and 3 had significantly higher levels of CCL20 in serum and CSF (8 pg/mL, 4–12 and median 3 pg/mL, 2–3, respectively, both *p* = 0.03). CCL20 was however not significantly higher when performing a covariate analysis with pleocytosis as a covariate. IL-17A levels in CSF correlated with pleocytosis (rho = 0.51, *p* < 0.0001).

Symptom duration before LP did not correlate with levels of cytokines/chemokines in serum or CSF in groups 1, 2 and 3 together. However, within group 2, duration of symptoms before LP correlated negatively with BAFF and CXCL13 in serum (rho = −0.57 and −0.58, respectively, both *p* < 0.01). When stratifying patient in groups 1, 2 and 3 according to duration of symptoms before LP, those with a shorter duration (<2 weeks, *n* = 49) had higher levels of BAFF in serum (median 764 ng/mL, 537–890, *p* = 0.002) compared to patients with longer symptom duration (*n* = 28) (556 ng/mL, 438–668).

**Fig. 2:**
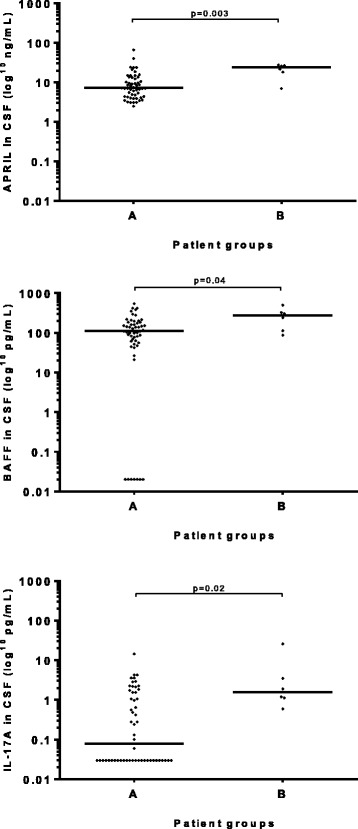
APRIL, BAFF and IL-17A levels in cerebrospinal fluid (CSF) in relation to duration of symptoms after treatment in group 1 (definite Lyme neuroborreliosis) and group 2 (possible Lyme neuroborreliosis with CSF pleocytosis) taken together. Group A: patients with time of recovery after treatment <3 months. Group B: patients with time of recovery after treatment >3 months. *Bars* represent the median cytokine level in each group

Regarding relation to disease course, patients in groups 1 and 2 were stratified according to time to recovery after treatment. Patients with shorter duration, group A (<3 months, *n* = 54) had lower levels of APRIL (*p* = 0.003), BAFF (*p* = 0.04) and IL-17A (*p* = 0.02) in CSF compared to patients with longer time of recovery, group B (>3 months, *n* = 6), (Fig. 2).

## Discussion

In this study, we showed that levels of several cytokines and chemokines related to Th17 and B cell immunity are raised in CSF from patients with LNB, strengthening the involvement of both Th17 and B cell immunity in LNB. Furthermore, we noted several relations to demographic and clinical parameters.

The lack of correlations between cytokine/chemokine levels in serum versus CSF indicates an intrathecal source of the cytokines and chemokines present in CSF, thus reflecting the pathological process in the CNS.

### B cell-related cytokines and chemokines

CXCL13 was significantly elevated in CSF of all LNB groups, in particular the pleocytosis groups 1 and 2, as compared to the non-LNB group. CXCL13 has been suggested as a diagnostic marker for acute LNB, and a majority of patients in groups 1 and 2, those with most probable acute LNB, showed raised CSF levels above 142 and 250 pg/mL, respectively, while no patients in group 3 had levels over 142 pg/mL, supporting CXCL13 as a diagnostic tool and corroborating several studies [4, 6–8, 21]. Patients in groups 1 and 2 with CSF CXCL13 levels below the cutoff values did not, however, differ in symptoms, duration of symptoms before LP or time to recovery after treatment compared to patients with higher levels of CXCL13. Other diagnoses than LNB cannot be completely ruled out in patients with CSF-CXCL13 levels below 142 pg/mL, especially in group 2, since this group only displayed CSF pleocytosis. We suggest that the LNB diagnosis in group 3 is questionable since these patients displayed no CSF pleocytosis and had CSF-CXCL13 levels below the suggested cutoff 142 pg/mL. Most of these patients reported symptoms less typical for LNB. Thus, the elevated AI could more likely reflect a previous infection, and other causes of their present symptoms are plausible. However, interestingly, slightly higher APRIL and CXCL13 CSF levels were found in this group compared with the non-LNB group, which may indicate a subtle ongoing B cell-related activity. On the other hand, CSF levels of CXCL1 were lower, strengthening group 3 as a different entity. Finally, regarding age, we corroborated previous findings [6] of increased levels of CXCL13 in children in both serum and CSF.

The involvement of B cell-related cytokines and chemokines in the pathogenesis of LNB is also supported by the raised CSF levels of both APRIL and BAFF in groups 1 and 2, the groups with most probable LNB. This is, to our knowledge, the first time elevated levels of APRIL has been reported in LNB patients, while BAFF has been studied previously [12]. The raised levels of APRIL and BAFF support the critical role of B cell activation and proliferation in LNB. However, moderately increased CSF levels of these cytokines were associated with shorter time to recovery (defined as <3 months), while higher levels were found in patients with longer time to recovery. Speculatively, moderate levels reflect an appropriate B cell response, while higher levels may reflect an over-shooting response mirroring or even contributing to more extensive CNS pathology. Increased levels of APRIL and BAFF have also been found in patients with multiple sclerosis [22] and systemic lupus erythematosus [11, 23], linked to antibody-mediated pathology and neuropsychiatric symptoms, respectively. Clearly, the role of APRIL and BAFF in LNB needs to be further elucidated, preferably in a prospective manner.

### Th17-related cytokines and chemokines

We found elevated CSF levels of IL-17A in LNB patients, which corroborates previous studies [13–15]. We here extended the concept of Th17 immunity by showing elevated levels of CXCL1 and CCL20, both induced by Th17 and involved in recruitment of neutrophils and Th17 cells, respectively. CXCL1 in CSF, was significantly raised in group 1, while CCL20 in CSF was significantly raised in all definite and possible LNB (groups 1, 2 and 3). This is, to our knowledge, the first study that shows these Th17-related markers in patients with LNB. IL-17A, CXCL1 and CCL20 have however been reported present in other Lyme borreliosis manifestations, such as Lyme arthritis (IL-17), erythema migrans and acrodermatitis chronica atrophicans (CXCL1 and CCL20) [24, 25]. In experimental studies, CXCL1 was shown to be produced by human astrocytes and brain microvascular endothelial cells in response to *B. burgdorferi* [26]. Interestingly, patients who recovered within 3 months after treatment had lower levels of IL-17A in CSF. Thus, high levels of IL-17A in CSF may be a prognostic marker and speculatively, a Th17 response could be involved in the pathogenesis of a delayed therapeutic response. In line with this notion, patients with prolonged symptoms after treatment of neurosyphilis had higher levels of IL-17A in CSF [27]. Further on, Th17 immunity has been linked to many autoimmune conditions, like rheumatoid arthritis [28] and psoriasis [29]. In CNS, Th17-related immune responses play a role in experimental autoimmune encephalomyelitis (EAE), an animal model for multiple sclerosis [30]. CCL20 can bind to the choroid plexus and lead Th17-related cells into CNS [30]. Our findings add further aspects of the Th17-related immune response in the pathogenesis of LNB and suggest that it may affect clinical course, although this needs to be confirmed.

There are some limitations of the current study. The retrospective design hampers clinical assessments. Another potential limitation is the lack of truly healthy controls, although the chosen group represents a clinically relevant reference group. Group 2, with mostly children, had CSF pleocytosis and characteristic symptoms of LNB, but other, foremost viral, infections cannot be completely ruled out, especially in cases with low CSF levels of CXCL13, since presence of neurotropic viruses was mostly not investigated. Regarding the EFNS guidelines, we note some limitations in the classification of possible LNB cases. According to the guidelines, patients corresponding to our groups 2 and 3 are classified as possible LNB, while we find important differences between the two groups in terms of clinical presentation and CSF findings, including cytokine and chemokine levels.

## Conclusions

We here demonstrate additional support for Th17 involvement in the intrathecal immune response in LNB as well as indications that high levels of IL-17A in CSF in the acute phase of the disease may be associated with slower recovery, hence proposing that IL-17A should be further evaluated as a possible biomarker for prognosis. Besides CXCL13, the B cell-related cytokines APRIL and BAFF are elevated in CSF from patients with LNB, and the levels could be associated with time to recovery after treatment.

## Abbreviations

AI: Antibody index
APRIL: A proliferation-inducing ligand
BAFF: B cell activating factor
CNS: Central nervous system
CSF: Cerebrospinal fluid
i.q.: Interquartile range
LNB: Lyme neuroborreliosis
LP: Lumbar puncture
Th cells: T helper cells
